# Engagement of a community advisory group to shape and build up participation in TB research

**DOI:** 10.5588/pha.23.0058

**Published:** 2024-03-01

**Authors:** L. H. Van, V. T. Nguyen, T. T. T. Le, T. N. T. Thanh, L. V. T. Nghi, N. H. Van, V. T. Q. Huong, M. Chambers, N. T. T. Thuong

**Affiliations:** ^1^ Oxford University Clinical Research Unit, Centre for Tropical Medicine, Ho Chi Minh City, Vietnam; ^2^ Centre for Tropical Medicine and Global Health, Nuffield Department of Medicine, Oxford University, Oxford, UK

**Keywords:** CAG, tuberculosis, community engagement, low- and middle-income countries, operational research

## Abstract

It is essential that communities at risk from TB are involved in TB research. Community advisory groups (CAGs) are one mechanism for involving communities in research and creating platforms for discussions between researchers and community members. We organised a CAG meeting with community members and people with lived experience in Ho Chi Minh City, Vietnam, to explore the community’s knowledge about TB and their perspectives on different diagnostic tests in Vietnam, a low-middle-income country with a high TB burden. Researchers shared basic information and addressed questions about TB. CAG members commented on preference of TB screening tests, and suggested that chest X-rays and blood tests were more acceptable than sputum tests because of the difficulty in sputum expectoration. In addition, clinical studies that required fewer visits to the hospitals would be preferred, even if this meant a greater reliance on blood sampling.

## Abstract (fr)

Il est essentiel que les communautés exposées au risque de TB soient impliquées dans la recherche sur la TB. Les groupes consultatifs communautaires (CAG, pour l’anglais « *community advisory groups* ») constituent un mécanisme permettant d'impliquer les communautés dans la recherche et de créer des plateformes de discussion entre les chercheurs et les membres de la communauté. Nous avons organisé une réunion du CAG avec des membres de la communauté et des personnes ayant une expérience vécue à Ho Chi Minh Ville, au Viêt Nam, afin d'explorer les connaissances de la communauté sur la TB et leurs perspectives sur les différents tests de diagnostic au Viêt Nam, un pays à revenu faible et moyen où la charge de la TB est élevée. Les chercheurs ont partagé des informations de base et répondu à des questions sur la TB. Les membres du CAG ont fait part de leur préférence pour les tests de dépistage de la TB et ont suggéré que les radiographies pulmonaires et les analyses de sang étaient plus acceptables que les tests d'expectoration en raison de la difficulté d'expectoration des crachats. En outre, les études cliniques qui nécessitent moins de visites dans les hôpitaux seraient préférées, même si cela implique une plus grande dépendance à l'égard des prélèvements sanguins.

TB remains a global public health crisis, with an estimated 10.6 million new cases and 1.3 million deaths worldwide in 2022.^1^ There has been a notable increase in advancements resulting from investments in TB research and drug development following the WHO's declaration of TB as a global emergency. There has also been growing involvement of the public through community engagement (CE) that can enhance the ethics and outcomes of research. Many research funders and stakeholders have become much more attentive in inquiries, engaging with participating communities in clinical research, especially randomised controlled trials.^2–5^ From the ethical and moral perspective, they contend that participants/patients are the ultimate end-users of medical research and should be involved in its production through such engagement activities.

CE is a process of working together with relevant stakeholders who share a common geographical location, interest or situation to address their health-related issues in the local context;^6,7^ soliciting community input, engaging them in decision-making, valuing their views and attitudes, and enabling individuals with TB to articulate their needs, preferences and concerns will provide meaningful insights for researchers. CE can also foster trust in research, enhance enrolment, and support post-trial implementation. A strong understanding of community needs can also enhance the acceptability, feasibility and effectiveness of new interventions in clinical trials.^5^

CE in TB research has recently been launched in a wide range of contexts, including 1) trial-specific initiatives (e.g., Mexican Consortium against TB,^8^ the Thibela TB trial,^9^ the UNITE4TB Community Advisory Group^10^); 2) research programme initiatives (e.g., Community Research Advisors Group to the TB Trials Consortium,^11^ the CE Programme of the TB Alliance,^12^ Community Advisory Board (CAB) of the South African TB Vaccine Initiative,^13^ Global CAB of the AIDS Clinical Trials Group^14^); and 3) global initiatives (e.g., the Community Task Force of the Stop TB Partnership,^15^ Global TB CAB,^16^ The Union's Community Advisory Panel^17^) (Table 1).^18^

**TABLE 1.** Overview of examples of community engagement activities in TB research.

Community engagement bodies	Objectives and activities
Trial-specific initiatives	
Mexican Consortium against TB^8^	This population-based prospective study examined DOTS in moderate rates of drug-resistant TB populations in Orizaba, Mexico, with the assistance of community-based health workers to facilitated passive case-finding and expanded the coverage of the WHO-recommended DOTS programme
Thibela TB trial^9^	This community-wide TB trial studied the preventive effect of TB treatment in gold mining settings in South Africa. It incorporated community engagement with a tailored communication strategy and participant advisory groups to facilitate and enhance enrolment
UNITE4TB Community Advisory Group (CAG)^10^	Established in 2022, the UNITE4TB CAG is a network of community representatives affected by TB from regions participating in the project. The CAG has contributed to all activities that involve communities and TB survivors, provided advice on the implementation of CE at trial sites and assisted in the development of educational materials with appropriate language to communicate with communities in understandable and adherent terms, to facilitate smooth trials with optimal recruitment and retention of trial participants
Research programme initiatives	
Community Research Advisors Group to the TB Trials Consortium (TBTC)^11^	The TBTC, a public–private partnership, involved central and local representatives of various diverse communities to advance research within local TB control programmes and to facilitate communication between local communities and trial investigators
Community Engagement Programme of the TB Alliance^12^	Launched in the REMox TB phase 3 trial, the TB Alliance CE Programme has engaged local communities through community advisory structures and offered ongoing technical support, direction and education for community engagement. These community outreach initiatives facilitate communication and collaboration among participants, community members and researchers, and give communities a voice in the research process
Community Advisory Board of the South African TB Vaccine Initiative (SATVI)^13^	First known for their BCG randomised clinical trial in 2000, SATVI has implemented various awareness events, public meetings, and cultural activities to disseminate TB education. SATVI has engaged and interacted with the community to increase awareness about TB and clinical research through World TB Day three peaks challenge, community Open Days, poster design competitions, pamphlets, newsletters, community radio, carina choice, and comic books
Global CAB of the AIDS Clinical Trials Group (ACTG)^14^	The ACTG is a network of researchers and community members with expertise in HIV/AIDS, TB, and emerging infectious diseases, established in 1987. The ACTG aims to align its research priorities with the needs of the populations it serves through the Global Community Advisory Board (GCAB), which operates at the international, national and local levels. The GCAB facilitates community outreach, education and involvement in research. It also enables affected communities to express their concerns, experiences and perspectives, and to ensure the use of appropriate language for study materials to enhance participant recruitment, retention and trial feasibility
Global initiatives	
Community Task Force, Stop TB Partnership^15^	The Task Force on Community Engagement of the Stop TB Partnership was established through community-based and community-led initiatives to incorporate community perspectives in TB diagnostics, implementation and access. The Community Task Force also strives to ensure that the perspectives of communities are taken into account in matters concerning TB care access, strengthening community systems, improving research literacy, and fostering meaningful engagement with TB communities
Global Tuberculosis Community Advisory Board (TB CAB)^16^	Established in 2011, the Global TB CAB is a network of researchers and community activists with expertise in TB and HIV. The TB CAB collaborates with, provides insightful input, advises clinical trial sponsors and investigators on various aspects of TB research, and promotes greater community engagement and awareness in the field
Community Advisory Panel, International Union Against Tuberculosis and Lung Disease (The Union)^17^	The Union’s Community Advisory Panel is a voluntary group of engaged civil society representatives, stakeholders, communications specialists, community actors and affected community representatives, such as medical practitioners and health activists, who contribute to and advise the Union’s board to best tailor and address the needs of affected communities

Community advisory groups (CAGs) are composed of small groups of individuals from the communities related to the research context.^18^ CAGs can create an opportunity for research participants or potential participants to strengthen their understanding of the research process and express their concerns. They also provide feedback on participants’ materials, including informed consent forms.^19^ There is a growing demand for good participatory practices in research and community engagement that are productive, acceptable, endorsed by the affected population, and aligned with the local context,^20^ and CAGs can provide platforms for this participation.

For more than three decades, CABs in low- and middle-income countries (LMICs) mainly concentrated on HIV-related studies, and CE in TB research received little or neglected attention.^5,21^ Vietnam, an LMIC, remains one of the high TB burden countries. In 2022, the estimated TB incidence in Vietnam was 176/100,000 population.^22^ There has been no published information on community engagement in Vietnam to assess the community’s perspectives of the various diagnostic tests for TB, including blood, sputum and urine, as well as their acceptability to participate in TB research prior to the clinical trials. TB diagnosis has been the largest gap in cascade of care, with almost 40% TB cases undiagnosed and therefore untreated.^23^ Obtaining sputum samples for TB diagnosis is sometimes challenging, especially in critical ill patients and children. The WHO and scientists have focused on developing novel non-sputum-based tests to improve TB diagnosis.^24^ Our inaugural CAG meeting aimed 1) to develop a TB-CAG of healthy volunteers and TB survivors to foster transparent dialogues between researchers and the public, and 2) to explore the participants’ perspectives on human sample donation for biomedical research.

## TB COMMUNITY ADVISORY GROUP SETTING

We worked with an existing community group – the Oxford University Clinical Research Unit (OUCRU) health research advisory board (HRAB), who have been supporting OUCRU’s research since 2021. We also invited additional community members with lived experience, including former pulmonary TB patients who had already finished TB treatment and household contacts of TB patients residing in Ho Chi Minh City (HCMC), Vietnam, from diverse socio-economic backgrounds (Table 2). A 2-hour meeting was held at the Oxford University Clinical Research Unit, HCMC (OUCRU-HCM), Vietnam. Our aim was to 1) explore their knowledge, beliefs, and perceptions about TB disease and research, and 2) to examine the acceptability of TB screening tests or participation in TB research.

**TABLE 2.** Baseline characteristics of included participants (*n* = 12).

Characteristics	*n (%)*
Age, years, min–max	16–64
Sex	
Female	8 (67.0)
Male	4 (33.0)
Education	
Higher education	6 (50.0)
Bachelor’s degree or equivalent	2 (16.7)
≤High school	4 (33.3)
Occupation	
Office staff	4 (33.3)
Engineer	1 (8.3)
Student	2 (16.7)
Other	5 (41.7)
Member of HRAB	9 (75.0)
Former TB patient	2 (16.7)
Lived with TB patients	1 (8.3)

HRAB = Health Research Advisory Board.

Before the meeting, OUCRU TB team built up a list of potential questions based on literature about common concerns and misconceptions about TB from the perspectives of the community and researchers in the TB field.^25–27^ We used a mixed-method approach to collect the participant responses, including closed and open-ended questions, and group discussions. First, to assess the participants’ knowledge about TB, we asked three open-ended questions, two group discussion questions, and four yes/no questions. Then, to measure the acceptability of TB screening tests or participation in TB research among participants, we administered six five-point Likert-scale questions,^28^ with a scale ranging from one (definitely do not accept) to five (totally accept and willing to do). Participants were instructed to choose only one option per question.

### Ethical approval and informed consent

Participation in the meeting was voluntary and participants retained the right to decline participation or withdraw at any time, as stated in the informed consent form or assent form for adolescents with parental consent. All participants provided written terms of reference and agreed to the publication of their discussion results. This meeting did not intervene, there were no risks to participants, and all personal information was anonymous. The meeting procedures followed the rules and regulations of OUCRU-HCM.

## CHARACTERISTICS OF TB-CAG PARTICIPANTS

There were a total of 12 participants with baseline characteristics as shown in Table 2. Our participants’ ages ranged from 16 to 64 years; 67.0% were female. Half of them had obtained a higher education degree or above (6/12), and a third were employed as office staff (4/12). Most participants had prior experience in attending CAG meetings (9/12, 75.0%). Two additional participants (17.0%) reported a history of previous TB disease, and one individual (8.0%) has lived with TB patients in their household.

### Knowledge about TB among TB-CAG participants

Table 3 presents an overview of the participant's level of knowledge concerning TB. Findings from the three distinct evaluation methods indicate that the majority of the participants exhibited a high level of consensus, providing reasonably accurate responses. To note, when asked about vaccination and infected organs, 100% of respondents answered correctly. Regarding the question on risks associated with sharing eating utensils and hereditary risk, while there was some variation in answers, respectively 83.3% and 90.9% of the responses were accurate.

**TABLE 3.** Participants’ knowledge about TB disease. **A)** Free text answers to open-ended questions (*n* = 12).

Responses	Frequency
What do you know about TB?	
TB is a contagious disease	3
TB caused by a bacteria called *M. tuberculosis*	1
People have *M. tuberculosis* in the body but are not infected with TB	1
TB meningitis is the most dangerous form of TB	1
There are many types of TB: pulmonary TB, bone TB, etc.	4
TB can cause death	1
Pham Ngoc Thach is the hospital specialising in TB in HCMC	1
There are two forms of TB: TB disease and latent TB infection	1
Drug-resistant TB and TB meningitis are challenging to cure	1
TB symptoms include fatigue, shortness of breath, etc.	1
Patients diagnosed with TB often do not have sufficient knowledge about TB and have not received prompt medical attention	1
How is TB transmitted?	
Transmission of TB through the respiratory tract	6
Transmission of TB via air droplets	1
Transmission of TB via contact	1
Transmission from people infected to others	1
How can TB be cured?	
TB can be cured	1
TB can be cured if patients receive the proper treatment	1
TB can be cured if patients adhere to treatment	3
Can be recurrent TB infection	1
Risk of death is high if TB patients do not receive treatment	1
TB can be cured if patients adhere to treatment and receive nutritional support	1

**B)** Open-ended questions discussed in the group discussion.		
	Group 1 (*n* = 6)	Group 2 (*n* = 6)
What symptoms will people with TB have?	Afternoon fever Cough Night sweats Weight loss Chest pain Pain is localised at the site of involvement (focal pain)	Persistent cough Fever (in the late afternoon) Loss of appetite Chest pain Shortness of breath Skin darkening (after taking drugs) Bone pain Headache, coma Fatigue Weight loss Haggard appearance
Who is susceptible to TB?	People have contact with TB patients Who lives with HIV Who are immunocompromised Who have long-COVID Healthcare workers in TB clinic Elderly, children, pregnant women	Healthcare workers Those who are immunocompromised People have contact with TB patients Household contact People working in closed rooms Active and passive smoker Who had not gotten a vaccination Who works in a polluted environment Who lives with HIV

	Yes	No
Yes/no questions (*n* = 12)	*n* (%)	*n* (%)
I have been vaccinated against TB (BCG); I will therefore not get TB again	0 (0.0)	12 (100.0)
TB only causes disease in the lungs, it does not affect any other organs	0 (0.0)	12 (100.0)
Sharing eating utensils can cause infection with TB	2 (16.7)	10 (83.3)
TB can be inherited^*^ (*n* = 11)	1 (9.1)	10 (90.9)

* One participant did not provide their response.

HCMC = Ho Chi Minh City.

### Acceptability of TB tests and research among TB-CAG participants

We asked participants to rank their acceptability of undergoing TB screening tests or participating in TB studies on a Likert scale (from 1 = “definitely do not accept” to 5 = “totally accept and willing to do”). The results of this ranking exercise are given in Table 4, which displays the level of acceptability for various routine screening tests.

**TABLE 4.** Acceptability of TB tests and research among participants.

	Likert scale				
	1	2	3	4	5
	*n* (%)	*n* (%)	*n* (%)	*n* (%)	*n* (%)
Acceptability of TB routine screening tests (*n* = 12)					
Chest X-ray	0 (0.0)	0 (0.0)	0 (0.0)	4 (33.3)	8 (66.7)
Sputum	2 (17.0)	2 (17.0)	3 (25)	1 (8.3)	4 (33.3)
Blood	1 (8.3)	0 (0.0)	0 (0.0)	5 (41.7)	6 (50.0)
Acceptability of participating in screening TB research (observational studies)					
The number of times to sample blood is more than usual^*^ (*n* = 10)	2 (20.0)	2 (20.0)	0 (0.0)	2 (20.0)	4 (40.0)
The amount of blood taken is more than usual (*n* = 12)	0 (0.0)	0 (0.0)	0 (0.0)	6 (50.0)	6 (50.0)
The number of times to collect sputum is more normally^*^ (*n* = 11)	2 (18.2)	4 (36.4)	1 (9.1)	0 (0.0)	4 (36.4)
Do you agree to participate in a TB screening study that requires additional, such as blood, sputum, or throat swab tests? *n* (%)					
Yes	9 (75.0)				
No	3 (25.0)				

* Some participants did not provide their responses.

Regarding TB routine screening tests, all 12 participants expressed agreement to have chest X-rays (CXRs) to screen for TB. Overall, participants were more likely to accept CXRs and blood-based tests over sputum-based tests as TB routine screening procedures. All 12 participants indicated their acceptance of providing extra volume of blood samples for TB research studies, in addition to the standard-of-care requirement. However, they expressed less enthusiasm for the prospect of increasing the frequency of sputum collection. Three-quarters of the participants voiced their willingness to participate in TB research.

### Perceived concerns and expectations from engaging in research

Utilising the modified socio-ecological model,^29^ we documented the perceived barriers and expectations among TB-CAG participants regarding their involvement in clinical studies or trials. The responses obtained were divided into two categories: individual and familial, as well as community, reflecting the distinct concerns and expectations of each group (Figure).

**FIGURE.**
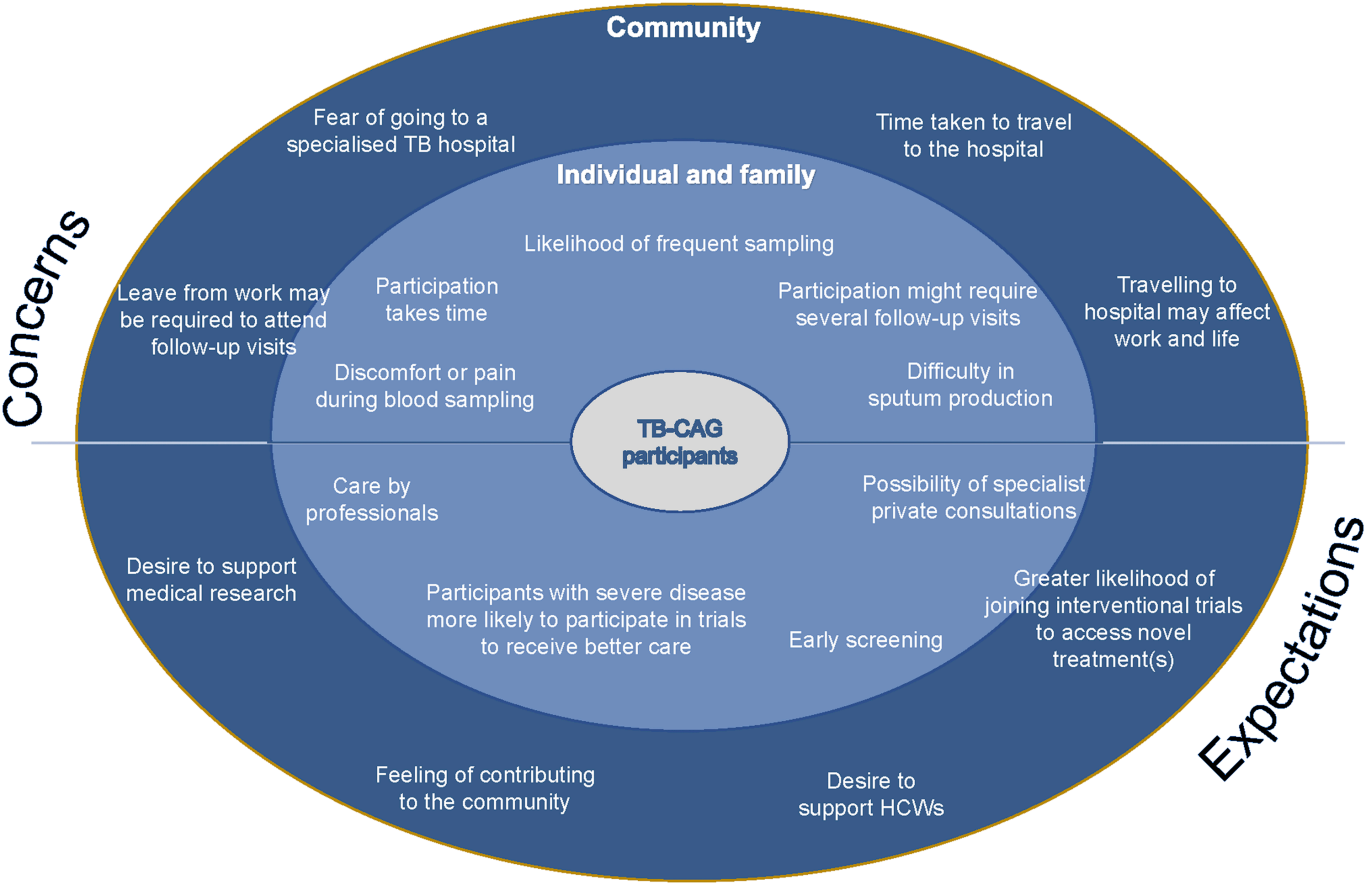
Concerns and expectations of TB-CAG participants in HCMC, Vietnam. TB-CAG = Tuberculosis Community Advisory Group; HCW = healthcare workers; HCMC = Ho Chi Minh City.

## THE FIRST TB-CAG FINDINGS AND FUTURE APPROACH

This TB-CAG meeting is the first meeting in our plan to build up and maintain regular activities to engage the community in TB research.

### General knowledge about TB disease

The members of this TB-CAG had good knowledge about TB, as most of them had already attended another TB public engagement activity held by the public and community engagement group in OUCRU. They knew correctly about what causes TB, how TB spreads, the symptoms and TB treatment. They understood the importance of getting diagnosed early. We provided them some additional information: TB diseases could happen in the lung (most common) but also in other organs such as the lymph nodes, brain, bone and skin. Pulmonary TB spreads through the air from those with active TB to others. Overall, without treatment, about 5–10% of infected persons will develop TB disease at some time in their lives. TB is curable but only if TB patients strictly adhere to and complete their treatment.

### Knowledge about TB infection

The TB-CAG also recognised the risks associated with contracting TB infection, such as exposure to TB patients, including those encountered while working in TB clinics and hospitals or having a family member diagnosed with TB; smoking, including active and passive smoking; due to immunosuppression; having HIV/AIDS, malnutrition, post-COVID syndrome; and being non-vaccinated. As a part of the meeting, we explained the benefits of the bacilli Calmette-Guérin vaccine in preventing disseminated and severe diseases in children, but also pointed out the fact that the vaccine cannot prevent pulmonary TB in adults.

### Acceptability of routine TB screening tests

Most participants were willing to have CXRs or blood tests as routine screening tests for TB (4–5 points per five scale of acceptance). Acceptability for a sputum-based test was scattered, from points 1 to 5 (per five scale of acceptance). CXRs and blood-based screening tests were more likely to be accepted as screening tests for TB than sputum-based screening tests. This was because CXRs and blood-based tests could be combined with regular health checks, and sputum could be hard to produce for some people.

### Acceptability of clinical TB studies

People had higher acceptability of TB studies that required more blood collected than studies that required more frequent visits to the hospitals. This was because TB clinic/hospital visits could increase exposure to TB patients and the risk of infection. Additional factors taken into account included screening/observational studies or interventional studies, as well as supplementary benefits like receiving closer care from study doctors compared to standard practice, and early diagnosis.

Although members of the TB-CAG might have higher than average education and better knowledge about TB than the community, their insight helped us to identify several challenges and barriers that may deter people from enrolling in clinical research in both interventional and non-interventional studies. Based on these insights the study team are incorporating their suggestions into future protocols the following measures: 1) reduction of the frequency of blood sampling, and 2) offering alternative locations for blood sampling so that participants, who are anxious about infection risk, do not have to enter the main TB hospital site. Comprising of participants from various backgrounds, this meeting provided insights into the community’s concerns and perspectives, and its expectations from scientists, and demonstrated the potential role of TB-CAG in community-oriented TB research in Vietnam.

Our first TB-CAG meeting had some limitations. First, the number of questions available to assess the level of acceptability among participants was limited, thereby impeding the comprehensive exploration of underlying beliefs. Second, the time limitations of a 2-hour meeting notably curtailed the discussion phase. This dynamic segment, where participants expressed their concerns and expectations, was relatively short to elicit a broader spectrum of responses. Finally, although the small number of participants was easier for interaction in group discussion, it hindered the representation of the diverse community in Vietnam.

This meeting established an initial CAG for TB research. In addition to discussing the overarching concept of research, potential topics such as consent forms, reimbursement procedures and consultations with the CAG on areas where community input is especially relevant will be included in the subsequent meeting. This TB-CAG will contribute to developing future outreach strategies because they can inform on what information they and their community want to receive and the appropriate approach method to be used. Our long-term goal is to create an environment where we can explore the community’s knowledge, attitudes, interests and concerns about TB to enable open discussion on specific TB research topics and disseminate research results to the community.

## CONCLUSIONS

Our TB-CAG meeting provided a valuable source of information for researchers, and built a bridge between researchers and community. The insights from the TB-CAG meeting provided the community perspective and concerns about TB research. Community concerns and expectations might help researchers and investigators understand how best to tailor and address the needs of the community, and thereby facilitate participant enrolment, retention and adherence.
